# Evaluation of Clinical Case Definitions for Respiratory Syncytial Virus Lower Respiratory Tract Infection in Young Children

**DOI:** 10.1093/jpids/piad028

**Published:** 2023-05-31

**Authors:** Janet A Englund, Rachel A Cohen, Veronique Bianco, Joseph B Domachowske, Joanne M Langley, Shabir A Madhi, Khalequ Zaman, Agustin Bueso, Ana Ceballos, Luis Cousin, Sanjay Gandhi, Olivier Gruselle, Lisa Jose, Nicola P Klein, Anthonet Koen, Thanyawee Puthanakit, Meng Shi, Peter Silas, Auchara Tangsathapornpong, Jamaree Teeratakulpisarn, Timo Vesikari, Gerco Haars, Amanda Leach, Sonia K Stoszek, Ilse Dieussaert

**Affiliations:** Seattle Children’s Research Institute/University of Washington, Seattle, Washington, USA; GSK, Rockville, Maryland, USA; GSK, Rockville, Maryland, USA; Department of Pediatrics, SUNY Upstate Medical University, Syracuse, New York, USA; Canadian Center for Vaccinology (Dalhousie University, IWK Health and Nova Scotia Health), Halifax, Nova Scotia, Canada; South African Medical Research Council Vaccines and Infectious Diseases Analytics Research Unit, University of the Witwatersrand, Johannesburg, South Africa; International Centre for Diarrheal Disease, Dhaka, Bangladesh; DEMEDICA, San Pedro Sula, Honduras; Instituto Médico Río Cuarto, Río Cuarto, Córdoba, Argentina; DEMEDICA, San Pedro Sula, Honduras; GSK, Mumbai, India; GSK, Wavre, Belgium; South African Medical Research Council Vaccines and Infectious Diseases Analytics Research Unit, University of the Witwatersrand, Johannesburg, South Africa; Kaiser Permanente Vaccine Study Center, Oakland, California, USA; South African Medical Research Council Vaccines and Infectious Diseases Analytics Research Unit, University of the Witwatersrand, Johannesburg, South Africa; Center of Excellence for Pediatric Infectious Diseases and Vaccines, Faculty of Medicine, Chulalongkorn University, Bangkok, Thailand; GSK, Rockville, Maryland, USA; Wee Care Pediatrics, Syracuse, Utah, USA; Faculty of Medicine, Thammasat University, Pathum Thani, Thailand; Faculty of Medicine, Khon Kaen University, Khon Kaen, Thailand; Nordic Research Network Oy, Tampere, Finland; GSK, Wavre, Belgium; GSK, Rockville, Maryland, USA; GSK, Rockville, Maryland, USA; GSK, Wavre, Belgium

**Keywords:** case definition, disease severity, hospitalization, infant, lower respiratory tract infections, newborn, respiratory syncytial virus

## Abstract

**Background:**

Various case definitions of respiratory syncytial virus lower respiratory tract infection (RSV-LRTI) are currently proposed. We assessed the performance of 3 clinical case definitions against the World Health Organization definition recommended in 2015 (WHO 2015).

**Methods:**

In this prospective cohort study conducted in 8 countries, 2401 children were followed up for 2 years from birth. Suspected LRTIs were detected via active and passive surveillance, followed by in-person clinical evaluation including single timepoint respiratory rate and oxygen saturation (by pulse oximetry) assessment, and nasopharyngeal sampling for RSV testing by polymerase chain reaction. Agreement between case definitions was evaluated using Cohen’s κ statistics.

**Results:**

Of 1652 suspected LRTIs, 227 met the WHO 2015 criteria for RSV-LRTI; 73 were classified as severe. All alternative definitions were highly concordant with the WHO 2015 definition for RSV-LRTI (*κ*: 0.95–1.00), but less concordant for severe RSV-LRTI (*κ*: 0.47–0.82). Tachypnea was present for 196/226 (86.7%) WHO 2015 RSV-LRTIs and 168/243 (69.1%) LRTI/bronchiolitis/pneumonia cases, clinically diagnosed by nonstudy physicians. Low oxygen saturation levels were observed in only 55/226 (24.3%) WHO 2015 RSV-LRTIs.

**Conclusions:**

Three case definitions for RSV-LRTI showed high concordance with the WHO 2015 definition, while agreement was lower for severe RSV-LRTI. In contrast to increased respiratory rate, low oxygen saturation was not a consistent finding in RSV-LRTIs and severe RSV-LRTIs. This study demonstrates that current definitions are highly concordant for RSV-LRTIs, but a standard definition is still needed for severe RSV-LRTI.

**Clinical trial registration:**

NCT01995175.

## Teaser

Respiratory syncytial virus (RSV) causes respiratory illnesses ranging from common cold to moderate and severe lung disease. Symptoms and methods used to identify/track RSV disease are not always the same in different settings. We compared how 3 definitions of RSV disease differ from a definition recommended in 2015 by the World Health Organization (WHO). We followed 2401 children from 8 countries from birth to 2 years and tracked all instances of RSV disease according to each definition. The number of RSV disease cases without severe symptoms was similar using all definitions (227 for the WHO, and between 226 and 236 for 3 other definitions). In contrast, numbers varied widely for children with severe symptoms (73 cases for the WHO, and between 23 and 145 cases for the other definitions). A universal definition of RSV disease and severity is needed to test and compare treatments and prevention measures against RSV.

## Graphical Abstract

**Graphical Abstract:**
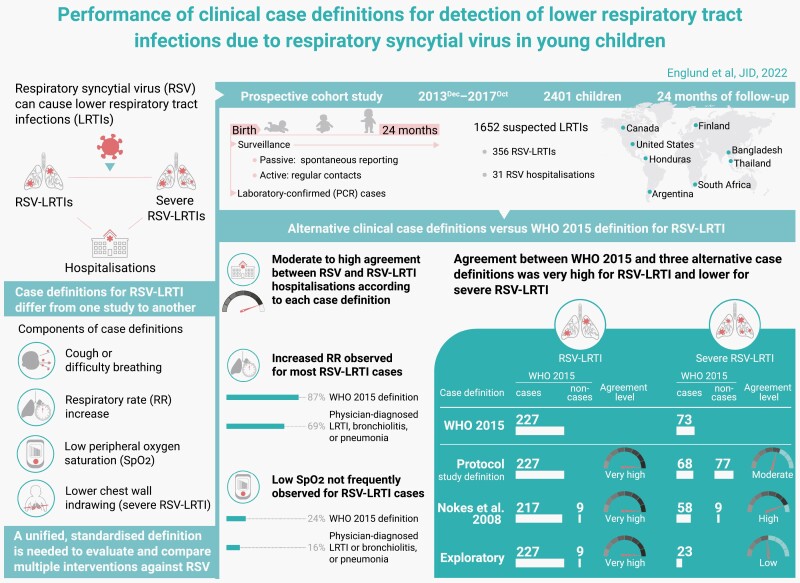

## Background

Globally, lower respiratory tract infections (LRTIs) remain one of the most common causes of morbidity and mortality, particularly in young children and low-income countries [1, 2]. Respiratory syncytial virus (RSV) is a leading cause of acute LRTI in children <6 months of age and a substantial burden on healthcare services worldwide [1, 3–5]. A meta-analysis of RSV global disease burden in children <5 years of age in 2019 estimated that 33.0 million RSV-LRTI cases, 3.6 million RSV-LRTI hospital admissions, and 101 400 RSV-attributable deaths (45% in infants <6 months) occur annually [5]. Many RSV-associated deaths occur in the community and may be overlooked by hospital-based surveillance, contributing to the high RSV mortality rates in low- and middle-income countries (LMICs) [6–11].

RSV disease burden estimates and cross-study comparisons are limited by differences in inclusion criteria, case ascertainment methodologies, and RSV diagnostic tests. Importantly, published studies of RSV disease burden or interventional trials have not used consistent case definitions for LRTI and severe LRTI. Consistent, standardized RSV-LRTI case definitions would allow comparisons of estimates between different populations and facilitate more efficient planning, implementation, and evaluation of RSV prevention programs [12]. A standardized definition will also strengthen a rigorous approach to the clinical development of RSV prevention interventions.

Starting in 1995, the World Health Organization (WHO) developed RSV-LRTI case definitions based on clinical symptoms and observations as part of the integrated management of childhood illness (IMCI) strategy to detect and treat acute LRTIs [13]. To evaluate interventions against RSV in clinical trials, the WHO revised these definitions in 2015 by incorporating oxygen saturation (SpO_2_) measurements (an indicator of hypoxemia) to diagnose and classify RSV-LRTI severity [12]. However, the use of pulse oximetry-measured SpO_2_ to assess severity of RSV disease is controversial, with no definitive documented cutoff values or validations [14–16].

We conducted a prospective international cohort study of infants from birth to 2 years of age to detect RSV-LRTI using active and passive surveillance, with laboratory confirmation of viral etiology. In this report, we assess the performance of the WHO 2015 case definitions and 3 other case definitions for RSV-LRTI.

## METHODS

### Study Design and Participants

We conducted this prospective, observational cohort study between December 2013 and October 2017 at study sites located in high-income countries (HICs; Canada, Finland, and the United States) and LMICs (Argentina, Bangladesh, Honduras, South Africa, and Thailand). The study design and eligibility criteria are described in detail elsewhere [17]. Briefly, children were enrolled at birth and followed during their first 2 years of life. LRTI cases were detected using active and passive surveillance (ie, regular site contacts vs. spontaneous reports). Here, we report the evaluation of 4 case definitions; other objectives have been described [17] or will be reported elsewhere.

We conducted the study in accordance with Good Clinical Practice, the Declaration of Helsinki, and all applicable regulatory requirements. The study protocol, subsequent amendments, and informed consent forms were reviewed and approved by national regulatory authorities and Institutional Review Boards/Institutional Ethics Committees at each site. The trial is registered on ClinicalTrials.gov (NCT01995175); the full study protocol is available at https://www.gsk-studyregister.com/en/trial-details/?id=200150.

### Procedures

The study procedures were described elsewhere [17]. Briefly, suspected LRTI was defined as RTI symptoms with potential LRTI involvement, including cough, runny nose, or blocked nose with any signs of breathing difficulty. Children with suspected LRTI identified through surveillance were examined in-person within 72 h to collect the following: temperature, respiratory rate (RR), SpO_2_, a nasopharyngeal swab sample, and all clinical symptoms. RR was measured manually, using a stethoscope and a counter/watch, for at least 60 s. SpO_2_ was determined using dedicated study-provided pulse oximeters, generally while the child was breathing room air.

RSV was detected and subtypes were characterized using an in-house reverse transcription-quantitative real-time polymerase chain reaction assay (RT-qPCR).

Parents/legally authorized representatives recorded any symptoms on diary cards for 14 days after identification of a suspected LRTI, or until symptom resolution (if >14 days). Each case was followed by study staff with subsequent regular contacts. Healthcare utilization during the entire episode was recorded. Any physician diagnosis of LRTI, pneumonia, or bronchiolitis during the episode, occurring outside the study and subsequent to the study examination visit, was recorded (hereafter denoted physician-diagnosed LRTI).

### Case Definitions

The 4 RSV-LRTI case definitions evaluated in this study are presented in Table 1. A case definition elaborated by the study sponsor in collaboration with experts (hereafter denoted protocol definition) and 1 used in surveillance in Kenya (Nokes definition) [18] were evaluated. An exploratory case definition not including chest indrawing to define severe disease was also evaluated following a 2014 change in the WHO IMCI guidance [19]. Any LRTI episode meeting criteria for a given case definition was counted as a case under that definition; all other episodes were considered noncases. Concordance of all 3 definitions with the WHO 2015 definition [12] was assessed.

**Table 1.** Case Definitions Used in the Study for RSV-LRTI

	WHO 2015 [12]	Protocol (Current Study)	Nokes et al. (2008) [18]	Exploratory
RSV-LRTI	▪ Child with history of cough or difficulty breathing	▪ Child with history of cough *or runny nose or blocked nose*	▪ Child with history of cough or difficulty breathing	▪ Child with history of cough or difficulty breathing
	▪ SpO_2_ <95% or RR increase	▪ SpO_2_ <95% or RR increase	▪ SpO_2_ <90% *accompanied by clinical diagnosis of LRTI/bronchiolitis*, or RR increase*, or lower chest wall indrawing*	▪ SpO_2_ <95%, or RR increase, *or lower chest wall indrawing*
	▪ RSV-positive by RT-qPCR	▪ RSV-positive by RT-qPCR	▪ RSV-positive by RT-qPCR	▪ RSV-positive by RT-qPCR
Severe RSV-LRTI	▪ Child with RSV-LRTI	▪ Child with RSV-LRTI	▪ Child with RSV-LRTI	▪ Child with RSV-LRTI
	▪ SpO_2_ <93% or lower chest wall indrawing	▪ SpO_2_ <92%, *or difficulty breathing (leading to irritability/agitation or lethargy/sleepiness),* or lower chest wall indrawing, *or reduced/no vocalization, or apnea >20 seconds, or cyanosis, or stop feeding well/dehydration*	▪ At least one of the following: SpO_2_*<90% accompanied by clinical diagnosis of LRTI/bronchiolitis* or lower chest wall indrawing	▪ SpO_2_ <93%

Abbreviations: LRTI, lower respiratory tract infection; RR, respiratory rate; RSV, respiratory syncytial virus; RT-qPCR, reverse transcription-quantitative polymerase chain reaction; SpO_2_, saturation of peripheral oxygen; WHO, World Health Organization.

*Notes*: RR increase was defined as ≥60/min (<2 months of age); ≥50/min (2–11 months of age); ≥40/min (12–24 months of age). RR, SpO_2_, and clinical symptoms were recorded by study staff at in-person visits conducted within 72 h from the identification of a new or worsened suspected LRTI case through active or passive surveillance.

### Statistical Analyses

The enrollment target was 2400 participants; sample size was determined as previously described [17]. Analyses were performed using data collected from children meeting all eligibility criteria until study completion or drop-out (eg, withdrawn consent, lost to follow-up, lack of compliance).

Clinical case definitions were compared with the WHO 2015 case definition based on RSV-LRTI or severe RSV-LRTI case status (case vs. noncase), considering all new suspected LRTI episodes, including recurrent events. Case definitions were also compared among the subset of RSV-positive suspected LRTI episodes. Agreement between RSV hospitalizations and RSV-LRTI hospitalizations (in-patient admission with or without intensive care) was also evaluated for each case definition. Concordance was estimated using Cohen’s kappa (*κ*) statistic [20, 21]; values from 0.60 to 0.79 indicated moderate agreement, from 0.80 to 0.90 strong agreement, and above 0.90 almost perfect agreement [22].

## RESULTS

### Study Population

Of 2402 children enrolled in the study, 2401 were included in the analyses; 1439 (59.9%) were from LMICs (Supplementary Figure 1) [17].

Baseline characteristics were previously described [17]. Briefly, most children (62.9%) were born during a period with high regional RSV transmission in each country (Supplementary Figure 1) and after 37 weeks of gestation (92.5%). Approximately half of children (49.5%) had mothers with a university education or higher; race distribution varied by country [17]. No children had an underlying condition associated with low SpO_2_.

### Agreement Between RSV-LRTI Definitions

In total, 1652 suspected LRTI episodes (565 first episodes of LRTI; Supplementary Table 1) were identified; 356 were RSV-positive. Per the WHO 2015 case definition, 227 episodes were RSV-LRTI cases and 1425 (including 129 RSV-positive episodes not meeting other defining criteria) were noncases; 73 were severe RSV-LRTI cases.

Around 85% of first episodes occurred in LMICs for both WHO 2015 RSV-LRTI (174 in LMICs, 32 in HICs) and severe RSV-LRTI (58 in LMICs, 11 in HICs). The proportion of WHO 2015 severe RSV-LRTIs among first episodes of WHO 2015 RSV-LRTI varied from 29.9% to 42.1% in LMICs and from 25.0% to 50.0% in HICs (Supplementary Table 1).

All 3 alternative definitions were highly concordant with the WHO 2015 definition (*κ* coefficients between 0.95 and 1.00; Supplementary Table 2). In a post hoc analysis considering only RSV-positive episodes, agreement with the WHO 2015 definition was similar for the protocol and exploratory definitions, but lower for the Nokes definition (Supplementary Table 2).

**Table 2.** Comparison Between WHO 2015 and Alternative Clinical Case Definitions for RSV-LRTI and Severe RSV-LRTI, All Suspected LRTI Episodes (*N* = 1652) Occurring in 2401 Children Followed Up for 2 Years From Birth

	WHO 2015 RSV-LRTI		
	Case	Noncase	*κ* (95% CI)
Protocol definition (current study)			
Case	227	0	1.00 (1.00–1.00)
Noncase	0	1425	
Nokes et al. 2008 definition			
Case	217	9	0.95 (0.93–0.97)
Noncase	10	1416	
Exploratory definition			
Case	227	9	0.98 (0.96–0.99)
Noncase	0	1416	
	WHO 2015 severe RSV-LRTI		
	Case	Noncase	*κ* (95% CI)
Protocol definition (current study)			
Case	68	77	0.60 (0.52–0.68)
Noncase	5	1502	
Nokes et al. (2008) definition			
Case	58	9	0.82 (0.75–0.89)
Noncase	15	1570	
Exploratory definition			
Case	23	0	0.47 (0.34–0.59)
Noncase	50	1579	

Abbreviations: case/noncase, any suspected LRTI episode meeting all criteria for a given case definition/all other suspected LRTI episodes; CI, confidence interval; LRTI, lower respiratory tract infection; RSV, respiratory syncytial virus; WHO, World Health Organization; κ, Cohen’s kappa coefficient.

*Note*s:Around 85% of first episodes of WHO 2015 RSV-LRTI and severe RSV-LRTI occurred in low- to middle-income countries (LMICs) (174 in LMICs and 32 in high-income countries [HICs] for WHO 2015 RSV-LRTI, and 58 in LMICs and 11 in HICs for WHO 2015 severe RSV-LRTI).

Of 73 WHO 2015 severe RSV-LRTI cases, 68 were also severe RSV-LRTI by the protocol definition; 1502 were noncases by both definitions. Overall, alternative case definitions for severe RSV-LRTI were less concordant than those for RSV-LRTI of any severity (κ coefficients between 0.47 and 0.82; Table 2). In a post hoc analysis among RSV-positive episodes only, agreement between alternative case definitions and the WHO 2015 definition was lower for the protocol definition and similar for other definitions (Supplementary Table 2).

RSV viral loads were highest among episodes meeting the WHO 2015 severe RSV-LRTI definition (Supplementary Figure 2).

### Comparison of RSV Hospitalizations to Case Definitions

The agreement between suspected RSV-LRTI hospitalizations and hospitalizations for each RSV-LRTI case definition is summarized in Supplementary Table 3. In total, 31 hospitalizations occurred among suspected RSV-LRTIs (Supplementary Table 1). Altogether, 8.8% of WHO 2015 RSV-LRTI and 16.4% of WHO 2015 severe RSV-LRTI episodes required hospitalization [17]. Among hospitalized children 0–11 months of age, the WHO 2015 case definition was met for 16 episodes; 10 were severe. Nine were RSV-positive but did not meet WHO 2015 LRTI criteria: 6 infants had breathing difficulty or wheezing (of whom 3 were dehydrated or not feeding well), and 1 had breath holding spells; in 2 cases hospital records were not located. Among children 12–23 months of age, 4 cases with hospitalization met the WHO 2015 RSV-LRTI criteria, of which 2 were severe.

### Evaluation of WHO 2015 Case Definition Components

The frequency of selected symptoms reported during the examination visits was similar for WHO 2015 RSV-LRTI and physician-diagnosed LRTI cases. For WHO 2015 severe RSV-LRTI episodes, this frequency tended to be higher for nostril flaring and irritability/agitation and was substantially higher for chest indrawing compared to WHO 2015 RSV-LRTI or physician-diagnosed LRTI episodes (Table 3).

**Table 3.** Frequency of Selected Symptoms Reported During the Examination Visit for Nonstudy Physician’s Diagnosis of LRTI, Bronchiolitis, or Pneumonia, WHO 2015 RSV-LRTI and WHO 2015 Severe RSV-LRTI Cases Occurring in 2401 Children Followed Up for 2 Years From Birth

	Physician’s Diagnosis of LRTI, Bronchiolitis, or Pneumonia (*N* = 243)		WHO 2015 RSV-LRTI (*N* = 226)		WHO 2015 Severe RSV-LRTI (*N* = 73)	
Symptom	*n*	% (95% CI)	n	% (95% CI)	n	% (95% CI)
Cough^a^	243	100 (98.5–100)	226	100 (98.4–100)	73	100 (95.1–100)
Blocked nose^a^	214	88.1 (83.3–91.9)	199	88.1 (83.1–92.0)	62	84.9 (74.6–92.2)
Runny nose^a^	224	92.2 (88.1–95.2)	211	93.4 (89.3–96.2)	68	93.2 (84.7–97.7)
Wheezing	187	77.0 (71.1–82.1)	182	80.5 (74.8–85.5)	63	86.3 (76.2–93.2)
Stridor	21	8.6 (5.4–12.9)	19	8.4 (5.1–12.8)	9	12.3 (5.8–22.1)
Flare of the nostrils	24	9.9 (6.4–14.3)	22	9.7 (6.2–14.4)	16	21.9 (13.1–33.1)
Irritability/agitation	72	29.6 (24.0–35.8)	66	29.2 (23.4–35.6)	30	41.1 (29.7–53.2)
Lethargy/sleepiness	17	7.0 (4.1–11.0)	14	6.2 (3.4–10.2)	8	11.0 (4.9–20.5)
Any chest indrawing	56	23.0 (17.9–28.9)	57	25.2 (19.7–31.4)	57	78.1 (66.9–86.9)
Reduced/no vocalization	42	17.3 (12.7–22.6)	45	19.9 (14.9–25.7)	18	24.7 (15.3–36.1)
Any apnea^a^	3	1.2 (0.3–3.6)	7	3.1 (1.3–6.3)	4	5.5 (1.5–13.4)
Cyanosis^a^	3	1.2 (0.3–3.6)	2	0.9 (0.1–3.2)	1	1.4 (0.0–7.4)
Stop feeding well	68	28.0 (22.4–34.1)	68	30.1 (24.2–36.5)	27	37.0 (26.0–49.1)
Dehydration	7	2.9 (1.2–5.8)	5	2.2 (0.7–5.1)	3	4.1 (0.9–11.5)
Fever (temperature >37.5°C)	62	25.5 (20.2–31.5)	63	27.9 (22.1–34.2)	24	32.9 (22.3–44.9)
Vomiting^a^	93	38.3 (32.1–44.7)	84	37.2 (30.9–43.8)	32	43.8 (32.2–55.9)
Diarrhea^a^	68	28.0 (22.4–34.1)	71	31.4 (25.4–37.9)	28	38.4 (27.2–50.5)

Abbreviations: CI, confidence interval; LRTI, lower respiratory tract infection; *n* (%), number (percentage) of cases with each symptom; *N*, total number of cases according to each definition; RSV, respiratory syncytial virus; WHO, World Health Organization.

*Notes*:

^a^Symptoms reported during the interview or on the diary card were also included. All suspected LRTI cases (new or worsened) were included in this analysis.

**Figure 1.**
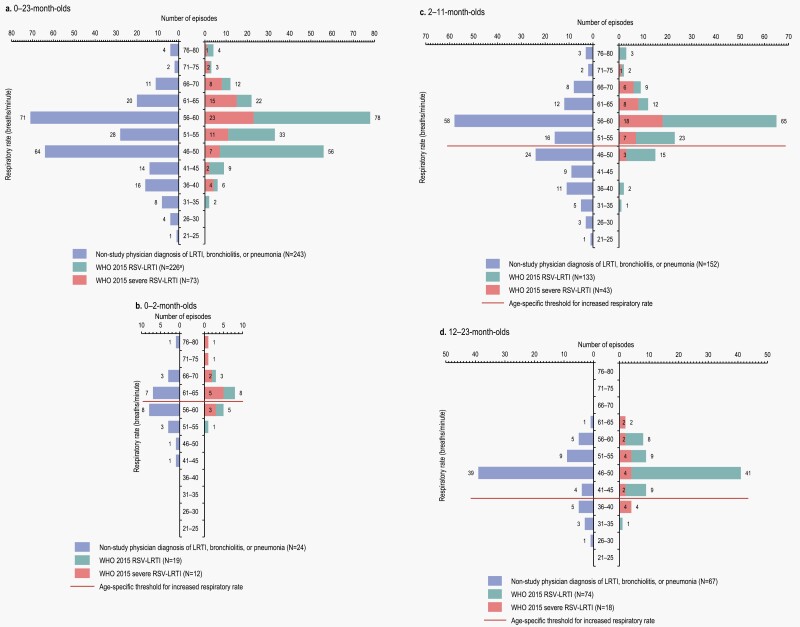
Frequency of respiratory rate, overall (a) and by age category (b–d), for WHO 2015 RSV-LRTI and severe RSV-LRTI and nonstudy physician diagnosis of LRTI, bronchiolitis, or pneumonia. LRTI, lower respiratory tract infection; *N*, number of cases according to each definition; RSV, respiratory syncytial virus; WHO, World Health Organization. ^a^One missing value.

The frequency of tachypnea and low SpO_2_ rates was also assessed. Among WHO 2015 RSV-LRTI episodes, data were available for 226; 1 episode (occurring in a 0–2 month-old child in Argentina) had not met LRTI criteria when the RSV-positive swab was collected (but met them later) and was therefore excluded from this analysis. Tachypnea was observed in most episodes (Figure 1). Overall, the RR was increased above the age-specific threshold for 196 of 226 (86.7%) WHO 2015 RSV-LRTI cases. For physician-diagnosed LRTI, a RR above the age-specific threshold was observed for 168 of 243 (69.1%) cases.

**Figure 2.**
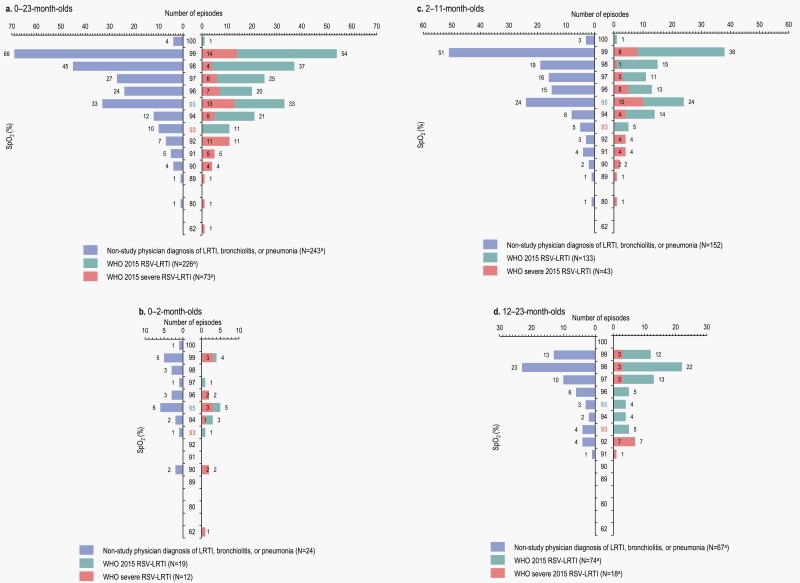
Oxygen saturation, overall (a) and by age category (b–d) for WHO 2015 RSV-LRTI and severe RSV-LRTI and nonstudy physician diagnosis of LRTI, bronchiolitis, or pneumonia. LRTI, lower respiratory tract infection; N, total number of cases according to each definition; RSV, respiratory syncytial virus; SpO_2_, saturation of peripheral oxygen; WHO, World Health Organization. The colored labels on the Y axis indicate the threshold for RSV-LRTI (95%) and severe RSV-LRTI (93%) for SpO_2_ rates according to the WHO 2015 case definitions.

Low SpO_2_ was not frequently identified in cases of WHO 2015 RSV-LRTI (Figure 2). SpO_2_ <95% was recorded for only 55 of 226 (24.3%) WHO RSV-LRTI cases and 40 of 243 (16.5%) physician-diagnosed LRTIs, while SpO_2_ <93% was recorded for 23 (10.2%) and 18 (7.4%) cases, respectively. Among the WHO 2015 severe RSV-LRTI cases, SpO_2_ was <95% in 38.4% of episodes and <93% in 31.5% of episodes. Overall and within each age group, SpO_2_ varied widely for severe RSV-LRTI episodes, ranging from 62% to 99% overall (0–2 months: 62–99%, 2–11 months: 80–99%, 12–23 months: 91–99%).

## DISCUSSION

Multiple interventions for the prevention of RSV infection in infants are in the late stages of clinical development [23, 24]. Consistent, validated, and simple definitions for reporting outcomes in RSV disease are critical as new methods of RSV disease prevention become available. This study is the first to assess the performance of several known case definitions in comparison to the WHO 2015 clinical case definition based on data obtained in a prospective longitudinal multicenter international study using PCR-based RSV detection and data collected across diverse geographic and economic settings.

Each of the explored 3 alternative case definitions was almost perfectly concordant with the WHO 2015 definition in identifying RSV-LRTI of any severity, undoubtedly reflecting the similarity between criteria used (SpO_2_ and tachypnea) across case definitions. Allowing runny or blocked nose as an alternative for history of cough in the criteria did not impact the detection of cases, as shown by the high agreement between the WHO 2015 and protocol definitions. The lowest concordance was observed for the Nokes definition, decreasing further when the analysis was repeated on RSV-positive cases only.

By contrast with cases of any severity, concordance between the WHO 2015 and the alternative case definitions was not high when comparing cases of severe RSV-LRTIs. This finding is important, as severe RSV-LRTI is often used as an endpoint in clinical trials. However, this was somewhat expected considering the higher variability of the criteria used to define severe disease across the different definitions, in particular the varying thresholds for SpO_2_ (<93% versus <92% vs. <90%). The protocol definition also included symptoms that were previously identified as clinical predictors for severe RSV requiring hospitalization, such as apnea [25, 26]. The Nokes definition showed the highest agreement with the WHO 2015 definition of severe RSV-LRTI. For the exploratory definition, lower chest wall indrawing was a criterion for RSV-LRTI but not of severe cases. Of note, lower chest wall indrawing is included in the WHO 2015 definition of severe RSV-LRTI for clinical trials [12]. However, in 2014 the WHO revised the 2005 pneumonia definitions to exclude it as a severity indicator, based on evidence that chest indrawing could safely be managed at home with oral antibiotics [19]. In a previous study, lower chest wall indrawing was not a clinical predictor for critical RSV-LRTI episodes (ie, requiring oxygen via mask, continuous or bilevel positive airway pressure, or mechanical ventilation) [27].

The role of individual components of the definition was also evaluated for the WHO 2015 case definition. Tachypnea was observed in most severe and nonsevere episodes. In contrast, most physician-diagnosed LRTI cases did not have tachypnea. Case definitions for LRTI rely on the RR criterion, which can raise challenges in a clinical setting, especially during the first few months of life. RRs can be influenced by many factors: ambient conditions, agitation, presence of fever, whether the child is awake or sleeping, body weight, or gender [28, 29]. Currently used methods are known to provide imprecise values [30]. In addition, in hospital settings there are various approaches to measure RR [31]; automatic methods and clinical observations may be more often employed in higher-resource settings while manual devices are used in low-resource settings (timers, counters), leading to potential differences in the accuracy of RR measurements.

Low SpO_2_ was not frequently identified in RSV-LRTI episodes. Importantly, there was a large overlap between the number of cases with the same SpO_2_ identified with the WHO 2015 case definitions and physician-diagnosed LRTI. Therefore, we could not identify a clear threshold for hypoxemia to suggest an improved definition of RSV-LRTIs or severe RSV-LRTIs in future interventional trials. In a previous study, SpO_2_ ≤90% at hospital admission was associated with an increased risk of critical LRTI due to RSV, although it was a nonspecific indicator when considered on its own (in the absence of other predictive clinical symptoms) [27]. The accuracy of SpO_2_ measurements has been shown to vary with race [32] and the SpO_2_ range, especially at values <90% [33]. For the same individual, values measured when lying at rest and sitting [34] or at different anxiety levels [35] may differ significantly. In addition, natural fluctuations in SpO_2_ occur over time [36]; therefore, using a single measurement in this study may have impacted our findings and led to lower numbers of cases with SpO_2_ <95%. Longitudinal or continuous SpO_2_ measurements might offer further insight into illness severity beyond a single measurement.

The use of SpO_2_ to clinically assess disease severity or to differentiate between severe and nonsevere LRTI has been debated and explored over recent decades. The WHO proposes thresholds of 93% for severe RSV-LRTI and 90% for very severe RSV-LRTI [12]. The American Academy of Pediatrics guidelines mention a cutoff of 90% for oxygen supplementation in bronchiolitis [14], while the United Kingdom NICE recommendations state that cases with SpO_2_ <92% should be referred for hospital emergency care [16]. However, a study assessing different hypoxemic targets in infants indicated no differences in the safety and clinical effectiveness for SpO_2_ targets of 90% and 94% in bronchiolitis management [15]. All guidelines recommend caution, highlighting the possibility of errors if relying on a single measurement or inappropriate instruments. Finally, standardized definitions for RSV illness severity should incorporate provisions for individuals with low baseline SpO_2_ measurements. Despite these potential issues, SpO_2_ for assessing disease severity is an objective measure that can be readily obtained in diverse clinical settings.

To date, a variety of LRTI case definitions are used as outcome measures for recent or ongoing clinical trials of preventive or treatment interventions against RSV (such as vaccines or monoclonal antibodies), which can lead to difficulty in comparing estimated vaccine or treatment efficacy. For instance, in a phase 3 trial evaluating the prevention of RSV-specific medically significant LRTI in infants whose mothers were vaccinated with a RSV-F-nanoparticle vaccine, evidence of medical significance was defined by the presence of hypoxemia (SpO_2_ <95% at sea level or <92% at an altitude of >1800 m) or tachypnea (≥70 breaths per minute at <60 days of age and ≥60 breaths per minute at ≥60 days of age) [37]. Other ongoing clinical trials are using different endpoints to assess vaccine efficacy, such as RSV-medically attended acute respiratory illness (NCT04520659), medically attended LRTI (NCT04424316), or medically assessed, RSV-associated LRTI (NCT04605159). These differences mean that vaccine efficacy estimates from these trials cannot be directly compared. For example, when considering tachypnea alone, over half of the RSV-LRTI cases identified in this analysis through the WHO 2015 case definition would not meet the case definition in the phase 3 trial for the maternal nanoparticle RSV-F vaccine. In the current study, we found that only 16.4% of severe RSV-LRTI episodes required hospitalization, varying largely by country [17], suggesting that hospitalization is also not a consistent indicator of RSV-LRTI severity. These observations underscore the need for a standardized case definition to support vaccine evaluation and decision-making related to new and evolving RSV prevention strategies.

As noted, our results do not provide insight to determine an optimal SpO_2_ threshold for severe RSV-LRTI. However, as SpO_2_ measurements varied widely among WHO 2015 severe RSV-LRTI cases, the results suggest that phase 4 studies conducted in settings with limited resources, where SpO_2_ is not readily available, could consider a case definition that omits SpO_2_ and relies on other indicators of severity. However, further investigation is needed as this study did not collect repeated SpO_2_ measurements.

This prospective, international study is one of the largest to date allowing comparison of case definitions with data collected through consistent methods across multi-national populations. However, the study has several limitations. The countries in this study may not be representative or generalizable globally. Despite efforts to standardize activities across all sites, there could still be potential differences in case ascertainment and follow-up and in site-specific return visit rates, which are necessary for staff to complete evaluations for suspected LRTIs. In addition, concordance between case definitions was not evaluated by economic setting (ie, LMICs vs. HICs) in post hoc analyses, due to an insufficient number of episodes in HICs. Of note, this study was designed only to investigate the agreement of alternative definitions with the WHO 2015 definition, and no analyses or modeling were conducted to validate a clinical RSV-LRTI case definition.

## CONCLUSION

Three alternative clinical RSV-LRTI case definitions showed excellent concordance with the WHO 2015 definition, but agreement was lower for severe RSV-LRTI. Fewer cases with increased RR were identified for physician-diagnosed LRTI than for WHO 2015 RSV-LRTI episodes; both definitions led to a similar, relatively low number of RSV-LRTI cases with low SpO_2_, even in severe cases. In contrast to increased RR, low SpO_2_ was not a consistent finding in RSV-LRTIs and severe RSV-LRTIs and did not appear concordant with other definition elements. Including more components might identify more RSV-LRTI cases. This study demonstrates that existing definitions are highly concordant for RSV-LRTI cases, but there is still a need to establish a standard definition of severe RSV-LRTI.

## Supplementary Material

piad028_suppl_Supplementary_Material

## Notes

***Financial support.*** This work was supported by GlaxoSmithKline Biologicals SA. GlaxoSmithKline Biologicals SA covered all costs associated with the development and publishing of the present manuscript.

***Potential conflicts of interest.*** R. A. C., V. B., S. G., O. G., M. S., and I. D. are GSK employees. G. H., A. L., and S. K. S. were GSK employees at the time the study was conducted. S. G., O. G., and S. K. S. have received personal fees and R. A. C. and I. D. have received other compensation from GSK, outside the submitted work. R. A. C., S. G., A. L., S. K. S., and I. D. hold shares in GSK. J. A. E., J. B. D., J. M. L., S. A. M., A. C., N. P. K., T. P., and T. V. have received research grants from GSK during the conduct of the study. J. A. E. has received grants from MedImmune (now AstraZeneca), Pfizer, Merck, and Novavax, and personal fees from Sanofi Pasteur, AstraZeneca, Meissa Vaccines, Pfizer, and Moderna, outside the submitted work. J. B. D. has also received research grants from MedImmune (now AstraZeneca), Pfizer, Novavax, and Diassess, outside the submitted work. J. M. L. also reports other compensation from Immunovaccine, grants from Novavax, Janssen, and Regeneron, outside the submitted work; in all cases these are paid to Dalhousie University. J. M. L. holds the Canadian Institutes of Health Research-GSK Chair in Pediatric Vaccinology at Dalhousie University. S. A. M. has received institution grants and personal fees from BMGF and GSK, and institution grants from Pfizer, Novavax, AstraZeneca, and Minervax. N. P. K. has received research grants from Pfizer, Sanofi Pasteur, Merck, GSK, and Protein Science (now Sanofi), outside the submitted work. T. V. has received research grants from VBI Vaccines and Janssen. K. Z., A. B., L. C. , L. J., A. K., P. S., A. T., and J. T.: No conflict.
