# Supplementary material for: Evaluation of Clinical Case Definitions for Respiratory Syncytial Virus Lower Respiratory Tract Infection in Young Children
Source: J Pediatric Infect Dis Soc. 2023 May 31;12(5):273–81. doi: 10.1093/jpids/piad028 (PMC10231393; doi:10.1093/jpids/piad028)
Supplement: piad028_suppl_Supplementary_Material [file piad028_suppl_supplementary_material.docx]

**Supplementary material**

# Table S1. Incidence rate of first episode of all cause-LRTI, WHO 2015 RSV-LRTI, WHO 2015 severe RSV-LRTI, and RSV hospitalisation, by age interval, overall and by country

|  |  | 0–11 months | | | |  | 12–23 months | | | |  | 0–23 months | | | |
| --- | --- | --- | --- | --- | --- | --- | --- | --- | --- | --- | --- | --- | --- | --- | --- |
| Country |  | N | n | PY | IR (95% CI), /100 PY |  | N | n | PY | IR (95% CI), /100 PY |  | N | n | PY | IR (95% CI), /100 PY |
| All cause LRTI | | | | | | | | | | | | | | | |
| Overall |  | 2401 | 414 | 2128.79 | 19.45 (17.62–21.41) |  | 1905 | 151 | 1746.82 | 8.64 (7.32–10.14) |  | 2401 | 565 | 3875.44 | 14.58 (13.40–15.83) |
| Argentina |  | 132 | 43 | 107.03 | 40.18 (29.08–54.12) |  | 86 | 18 | 72.50 | 24.83 (14.72–39.24) |  | 132 | 61 | 179.58 | 33.97 (25.98–43.63) |
| Bangladesh |  | 100 | 50 | 67.59 | 73.98 (54.91–97.53) |  | 49 | 14 | 41.02 | 34.13 (18.66–57.26) |  | 100 | 64 | 108.67 | 58.90 (45.36–75.21) |
| Canada |  | 143 | 17 | 134.22 | 12.67 (7.38–20.28) |  | 124 | 25 | 100.82 | 24.80 (16.05–36.61) |  | 143 | 42 | 234.99 | 17.87 (12.88–24.16) |
| Finland |  | 490 | 39 | 462.67 | 8.43 (5.99–11.52) |  | 442 | 10 | 433.57 | 2.31 (1.11–4.24) |  | 490 | 49 | 896.22 | 5.47 (4.04–7.23) |
| Honduras |  | 298 | 147 | 216.30 | 67.96 (57.42–79.88) |  | 150 | 29 | 130.54 | 22.21 (14.88–31.90) |  | 298 | 176 | 346.85 | 50.74 (43.52–58.82) |
| South Africa |  | 585 | 75 | 518.84 | 14.46 (11.37–18.12) |  | 467 | 27 | 420.08 | 6.43 (4.24–9.35) |  | 585 | 102 | 938.79 | 10.87 (8.86–13.19) |
| Thailand |  | 324 | 25 | 308.70 | 8.10 (5.24–11.96) |  | 294 | 23 | 273.74 | 8.40 (5.33–12.61) |  | 324 | 48 | 582.28 | 8.24 (6.08–10.93) |
| United States |  | 329 | 18 | 313.45 | 5.74 (3.40–9.08) |  | 293 | 5 | 274.55 | 1.82 (0.59–4.25) |  | 329 | 23 | 588.07 | 3.91 (2.48–5.87) |
| WHO 2015 RSV-LRTI | | | | | | | | | | | | | | | |
| Overall |  | 2401 | 147 | 2277.55 | 6.45 (5.45–7.59) |  | 2167 | 59 | 2056.43 | 2.87 (2.18–3.70) |  | 2401 | 206 | 4333.84 | 4.75 (4.13–5.45) |
| Argentina |  | 132 | 17 | 121.75 | 13.96 (8.13–22.36) |  | 112 | 8 | 103.29 | 7.75 (3.34–15.26) |  | 132 | 25 | 225.10 | 11.11 (7.19–16.39) |
| Bangladesh |  | 100 | 18 | 88.75 | 20.28 (12.02–32.05) |  | 81 | 1 | 80.22 | 1.25 (0.03–6.95) |  | 100 | 19 | 169.06 | 11.24 (6.77–17.55) |
| Canada |  | 143 | 6 | 139.50 | 4.30 (1.58–9.36) |  | 135 | 4 | 123.80 | 3.23 (0.88–8.27) |  | 143 | 10 | 263.25 | 3.80 (1.82–6.99) |
| Finland |  | 490 | 10 | 477.46 | 2.09 (1.00–3.85) |  | 469 | 2 | 464.37 | 0.43 (0.05–1.56) |  | 490 | 12 | 941.82 | 1.27 (0.66–2.23) |
| Honduras |  | 298 | 64 | 260.31 | 24.59 (18.93–31.40) |  | 232 | 23 | 217.86 | 10.56 (6.69–15.84) |  | 298 | 87 | 478.19 | 18.19 (14.57–22.44) |
| South Africa |  | 585 | 17 | 553.15 | 3.07 (1.79–4.92) |  | 523 | 3 | 485.45 | 0.62 (0.13–1.81) |  | 585 | 20 | 1038.44 | 1.93 (1.18–2.97) |
| Thailand |  | 324 | 8 | 317.70 | 2.52 (1.09–4.96) |  | 311 | 15 | 296.01 | 5.07 (2.84–8.36) |  | 324 | 23 | 613.55 | 3.75 (2.38–5.62) |
| United States |  | 329 | 7 | 318.93 | 2.19 (0.88–4.52) |  | 304 | 3 | 285.44 | 1.05 (0.22–3.07) |  | 329 | 10 | 604.43 | 1.65 (0.79–3.04) |
| WHO 2015 severe RSV-LRTI | | | | | | | | | | | | | | | |
| Overall |  | 2401 | 54 | 2326.41 | 2.32 (1.74–3.03) |  | 2260 | 15 | 2173.39 | 0.69 (0.39–1.14) |  | 2401 | 69 | 4499.70 | 1.53 (1.19–1.94) |
| Argentina |  | 132 | 7 | 126.31 | 5.54 (2.23–11.42) |  | 122 | 2 | 115.84 | 1.73 (0.21–6.24) |  | 132 | 9 | 242.23 | 3.72 (1.70–7.05) |
| Bangladesh |  | 100 | 7 | 96.26 | 7.27 (2.92–14.98) |  | 92 | 1 | 91.15 | 1.10 (0.03–6.11) |  | 100 | 8 | 187.51 | 4.27 (1.84–8.41) |
| Canada |  | 143 | 4 | 140.26 | 2.85 (0.78–7.30) |  | 137 | 1 | 127.66 | 0.78 (0.02–4.36) |  | 143 | 5 | 267.87 | 1.87 (0.61–4.36) |
| Finland |  | 490 | 1 | 482.4 | 0.21 (0.01–1.15) |  | 478 | 2 | 473.37 | 0.42 (0.05–1.53) |  | 490 | 3 | 955.76 | 0.31 (0.06–0.92) |
| Honduras |  | 298 | 25 | 280.60 | 8.91 (5.77–13.15) |  | 271 | 1 | 268.56 | 0.37 (0.01–2.07) |  | 298 | 26 | 549.19 | 4.73 (3.09–6.94) |
| South Africa |  | 585 | 4 | 559.48 | 0.71 (0.19–1.83) |  | 536 | 2 | 498.64 | 0.40 (0.05–1.45) |  | 585 | 6 | 1057.97 | 0.57 (0.21–1.23) |
| Thailand |  | 324 | 3 | 320.10 | 0.94 (0.19–2.74) |  | 316 | 6 | 308.11 | 1.95 (0.71–4.24) |  | 324 | 9 | 628.05 | 1.43 (0.66–2.72) |
| United States |  | 329 | 3 | 321.01 | 0.93 (0.19–2.73) |  | 308 | 0 | 290.05 | 0.00 (0.00–1.27) |  | 329 | 3 | 611.12 | 0.49 (0.10–1.43) |
| RSV hospitalisation | | | | | | | | | | | | | | | |
| Overall |  | 2401 | 25 | 2341.24 | 1.07 (0.69–1.58) |  | 2290 | 6 | 2206.44 | 0.27 (0.10–0.59) |  | 2401 | 31 | 4547.59 | 0.68 (0.46–0.97) |
| Argentina |  | 132 | 3 | 128.54 | 2.33 (0.48–6.82) |  | 126 | 0 | 121.11 | 0.00 (0.00–3.05) |  | 132 | 3 | 249.72 | 1.20 (0.25–3.51) |
| Bangladesh |  | 100 | 4 | 96.24 | 4.16 (1.13–10.64) |  | 95 | 0 | 94.26 | 0.00 (0.00–3.91) |  | 100 | 4 | 190.60 | 2.10 (0.57–5.37) |
| Canada |  | 143 | 1 | 142.13 | 0.70 (0.02–3.92) |  | 140 | 0 | 130.76 | 0.00 (0.00–2.82) |  | 143 | 1 | 272.84 | 0.37 (0.01–2.04) |
| Finland |  | 490 | 7 | 478.48 | 1.46 (0.59–3.01) |  | 473 | 0 | 469.53 | 0.00 (0.00–0.79) |  | 490 | 7 | 948.00 | 0.74 (0.30–1.52) |
| Honduras |  | 298 | 3 | 294.85 | 1.02 (0.21–2.97) |  | 293 | 0 | 291.46 | 0.00 (0.00–1.27) |  | 298 | 3 | 586.34 | 0.51 (0.11–1.50) |
| South Africa |  | 585 | 5 | 558.87 | 0.89 (0.29–2.09) |  | 535 | 0 | 498.34 | 0.00 (0.00–0.74) |  | 585 | 5 | 1057.06 | 0.47 (0.15–1.10) |
| Thailand |  | 324 | 1 | 321.08 | 0.31 (0.01–1.74) |  | 318 | 6 | 308.93 | 1.94 (0.71–4.23) |  | 324 | 7 | 629.85 | 1.11 (0.45–2.29) |
| United States |  | 329 | 1 | 321.06 | 0.31 (0.01–1.74) |  | 310 | 0 | 292.05 | 0.00 (0.00–1.26) |  | 329 | 1 | 613.18 | 0.16 (0.00–0.91) |

LRTI, lower respiratory tract infection; WHO, World Health Organization; RSV, respiratory syncytial virus; N, total number of children; n, number of first episodes (in all children in a given age group); PY, person-years; IR, incidence rate; CI, confidence interval.

**Table S2.** Comparison between WHO 2015 and alternative clinical case definitions for RSV-LRTI and severe RSV-LRTI, all RSV-positive episodes (N=356) occurring in 2401 children followed up for 2 years from birth

|  | WHO 2015 RSV-LRTI | | |
| --- | --- | --- | --- |
|  | Case | Non-case | κ (95% CI) |
| Protocol definition (current study) | | | |
| Case | 227 | 0 | 1.00 (1.00–1.00) |
| Non-case | 0 | 129 |  |
| Nokes et al. 2008 definition | | | |
| Case | 217 | 9 | 0.88 (0.83–0.94) |
| Non-case | 10 | 120 |  |
| Exploratory definition | | | |
| Case | 227 | 9 | 0.94 (0.91–0.98) |
| Non-case | 0 | 120 |  |
|  | WHO 2015 severe RSV-LRTI | | |
|  | Case | Non-case | κ (95% CI) |
| Protocol definition (current study) | | | |
| Case | 68 | 77 | 0.48 (0.40–0.57) |
| Non-case | 5 | 206 |  |
| Nokes et al. 2008 definition | | | |
| Case | 58 | 9 | 0.79 (0.71–0.87) |
| Non-case | 15 | 274 |  |
| Exploratory definition | | | |
| Case | 23 | 0 | 0.42 (0.30–0.54) |
| Non-case | 50 | 283 |  |

WHO, World Health Organization; RSV, respiratory syncytial virus; LRTI, lower respiratory tract infection; κ, Cohen’s kappa coefficient; CI, confidence interval.

**Table S3.** Comparisons between RSV hospitalisations and RSV-LRTI hospitalisations or severe RSV-LRTI hospitalisations according to the different case definitions used in the study, all suspected LRTI episodes (N=1652) occurring in 2401 children followed up for 2 years from birth

|  | RSV hospitalisation | | | Percentage of hospitalised cases meeting each definition |
| --- | --- | --- | --- | --- |
| RSV-LRTI hospitalisation | Case | Non-case | κ (95% CI) |  |
| WHO 2015 definition |  |  |  |  |
| Case | 20 | 0 | 0.78 (0.66–0.91) | 64.5% |
| Non-case | 11 | 1621 |  |  |
| Protocol definition |  |  |  |  |
| Case | 20 | 0 | 0.78 (0.66–0.91) | 64.5% |
| Non-case | 11 | 1621 |  |  |
| Nokes et al. 2008 definition |  |  |  |  |
| Case | 23 | 0 | 0.85 (0.75–0.95) | 74.2% |
| Non-case | 8 | 1621 |  |  |
| Exploratory definition |  |  |  |  |
| Case | 23 | 0 | 0.85 (0.75–0.95) | 74.2% |
| Non-case | 8 | 1621 |  |  |
| Severe RSV-LRTI hospitalisation |  |  |  |  |
| WHO 2015 definition |  |  |  |  |
| Case | 12 | 0 | 0.55 (0.37–0.73) | 38.7% |
| Non-case | 19 | 1621 |  |  |
| Protocol definition |  |  |  |  |
| Case | 17 | 0 | 0.70 (0.56–0.85) | 54.8% |
| Non-case | 14 | 1621 |  |  |
| Nokes et al. 2008 definition |  |  |  |  |
| Case | 13 | 0 | 0.59 (0.41–0.76) | 41.9% |
| Non-case | 18 | 1621 |  |  |
| Exploratory definition |  |  |  |  |
| Case | 6 | 0 | 0.32 (0.13–0.51) | 19.3% |
| Non-case | 25 | 1621 |  |  |

RSV, respiratory syncytial virus; LRTI, lower respiratory tract infection; CI, confidence interval; κ, Cohen’s kappa coefficient; WHO, World Health Organization.

**Figure S1.** Participant flowchart, overall and by country, and RSV transmission periods in each country


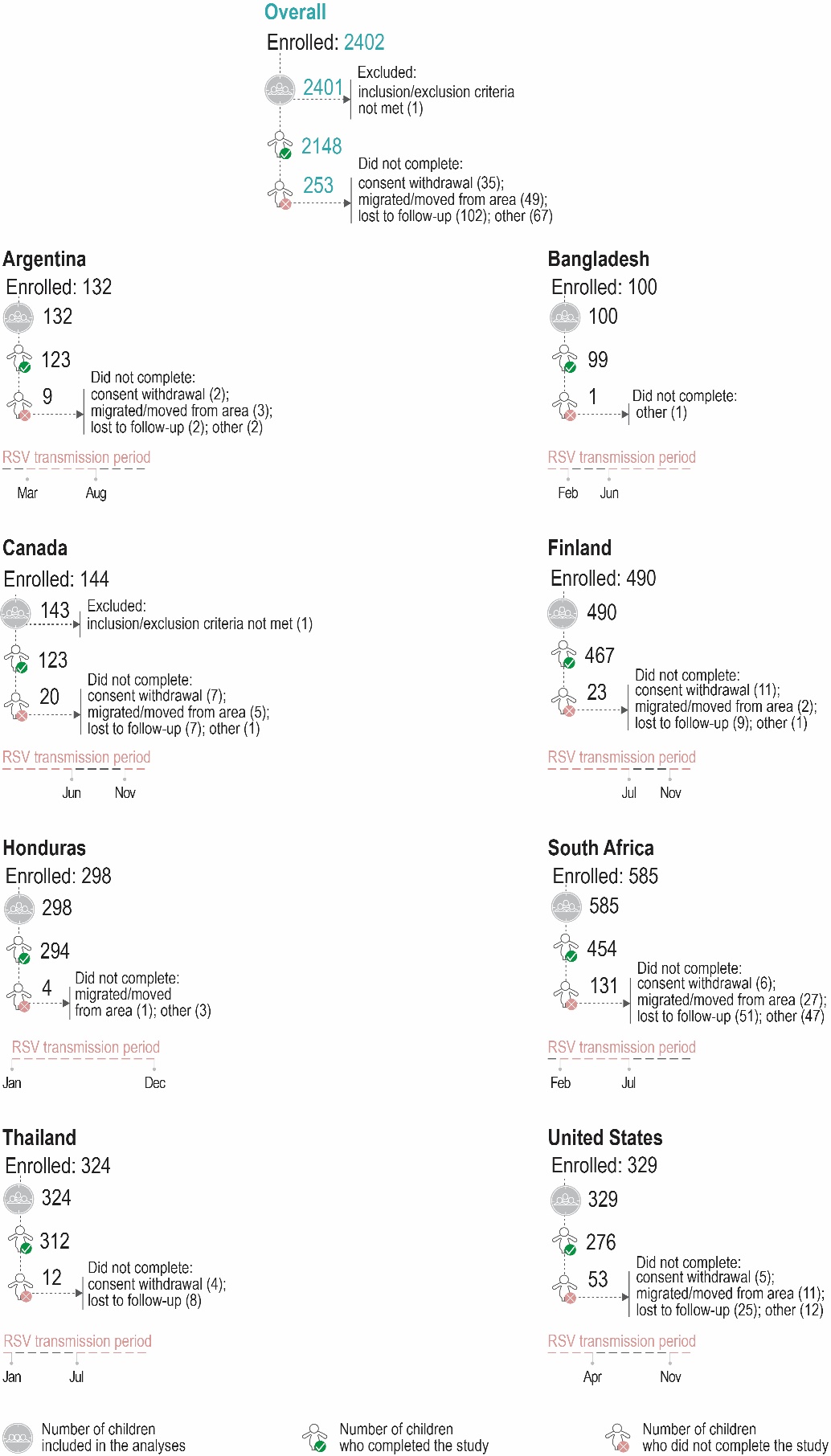


RSV, respiratory syncytial virus.

Note: The RSV transmission period in each country was defined based on the actual observed RSV season during the period of surveillance for each country. For countries such as Canada, the United States, and Finland which had well-established RSV surveillance systems in place near the study sites, the actual reported RSV season start and stop dates (by month) were used to define the RSV transmission period. If the RSV season was longer in one year than the other and >5% of children for that country were exposed to the longer season, then the longer season was considered. In countries without a robust RSV surveillance system, the RSV transmission period was established through the identification of at least one RSV case in each month defined as being within the RSV season. In order for the month to be counted as within the RSV season for that country, the month had to be adjacent to at least one other month that similarly met the definition for being within the RSV season.

# Figure S2. Total RSV viral load during all RSV episodes classified according to the WHO severity scale by non-overlapping disease severity, overall (a) and by age interval (b, c)


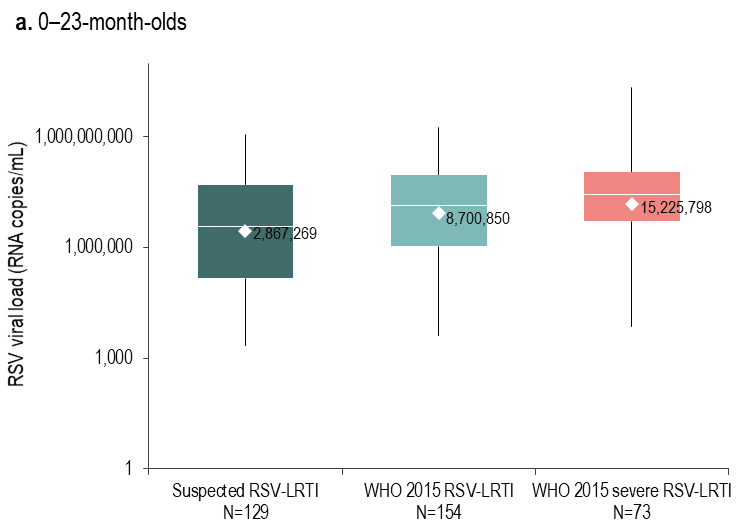


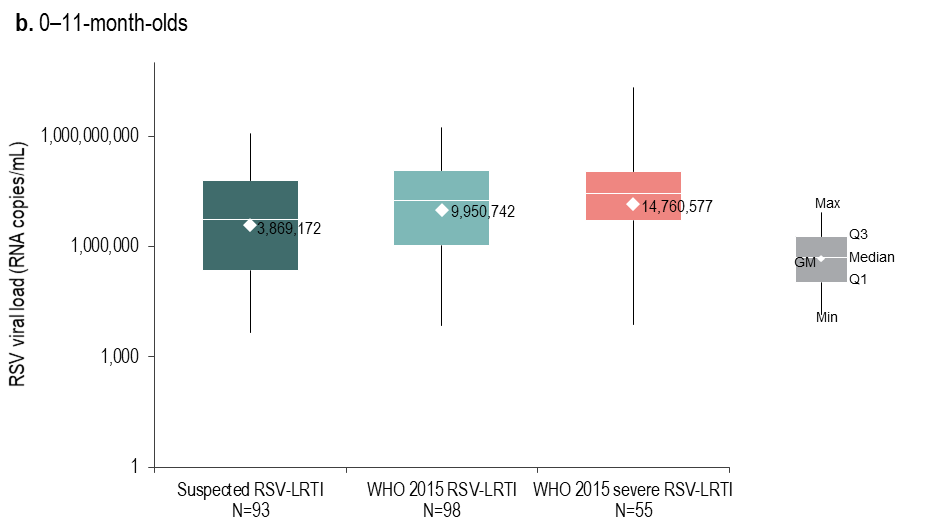


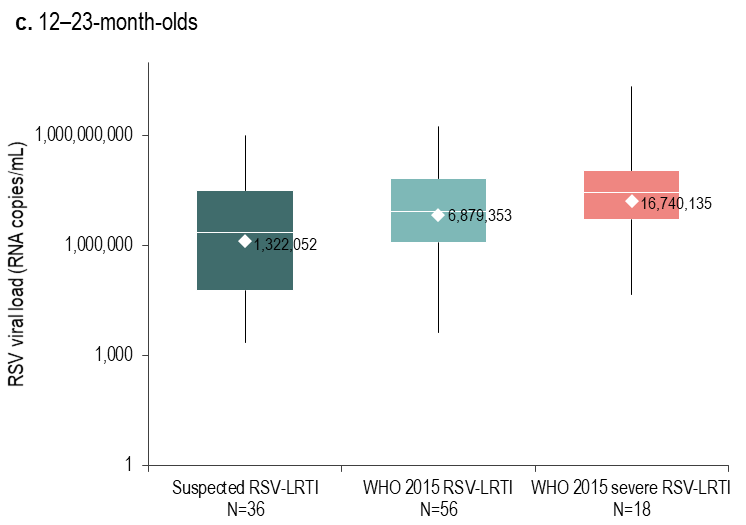


RSV, respiratory syncytial virus; WHO, World Health Organization; RNA, ribonucleic acid; LRTI, lower respiratory tract infection; Max, maximum; Q, quartile; GM, geometric mean; Min, minimum; N, total number of episodes meeting the case definition.

Notes: Analyses of the total RSV viral load during all RSV episodes were descriptive and were performed based on non-overlapping disease severity between suspected RSV-LRTI, WHO 2015 RSV-LRTI and WHO 2015 severe RSV-LRTI episodes (e.g., WHO 2015 severe RSV-LRTI episodes were not included in WHO 2015 RSV-LRTI episodes). The total RSV viral load was computed by adding the viral load from RSV-A and RSV-B tests (same swab). The median, minimum, maximum (with quartiles) and geometric means of viral load were calculated. If multiple swabs were collected per episode, the swab with the highest viral load was considered for this analysis. The analysis was not adjusted for other potentially relevant factors such as time of swab collection from symptom onset.
